# Registration: Its Limitations and a Plan of Campaign

**Published:** 1917-03-17

**Authors:** 


### SOME NURSING PROBLEMS IN IRELAND.

### Registration: its Limitations and a Plan of Campaign.

While nursing affairs in Ireland are simmering, and before any schemes that may be in contemplation take definite shape, may I take the opportunity of submitting a few observations for the consideration of your readers? As I stated last week, I am not a believer in the omnipotence of a Register of Trained Nurses, although I freely admit that it has its advantages, and is well worth striving after. Unless the forming of a Register is accompanied by such legislation as will put out of business all except trained nurses, it cannot accomplish a great deal for the nurse's material benefit. And if it can I should like somebody to explain exactly how. In discussing registration far too much is taken for granted, and it is often assumed that the conditions which govern the practice of medicine apply equally to nursing, which is obviously a fallacy. What I wish now to state is that no scheme is of the smallest value to any nurse, no matter from what source it emanates, unless it brings material benefit in its train. This may appear to be a platitude, but, despite the fact that it is elementary, I do not believe that it is generally realised or understood. Poverty of professional benefits is a distinguishing feature of the Irish nursing world. As probationers, nurses are penalised; when they are qualified they are badly paid. They are without holiday homes or railway privileges, and they have no pension scheme of their own. In a word, they are in a back street. Lack of organisation and sound leadership kept the midwifery section out of the English Bill when it was passing through Parliament. Then, as now, the profession here thought that they were going to be "done," with the result that while they dallied England, Scotland, and Wales have travelled far on the road of progress.

### A Plan of Campaign.

Everybody in Ireland knows what is meant by the term "plan of campaign." It was an effective scheme or method (never mind its ethics) devised by Irish farmers many years ago to secure improved conditions of land tenure and a reduction of rent. May I with all frankness ask those excellent persons who are pioneering the cause of nurses, in Great Britain or here, if they have got a plan of campaign, and if they can define its purpose with the same precision that the Irish farmers defined theirs? There is much talk here about an Irish diploma for nurses, a diploma with a medical imprimatur or something of a kindred nature. When this is granted to a thousand, or to seven thousand nurses, will they become any better off? Will they have an extra sovereign in their pockets, or an hour less duty to perform in the week? True, they may possess another scrap of paper, but this will not pass anywhere as current coin, or possess any purchasing power. If there are three thousand, or thirteen thousand, unqualified women working in Ireland as nurses now, they will still be working. The qualifications and eligibility of trained and untrained nurses will be, as they say in the Army, "as you were."

### The Workers' Wants.

May I be pardoned for suggesting that this aspect of the case has received scarcely sufficient consideration from the promoters of the Royal British College of Nursing? The mass of the nurses have not before them a plan of campaign with well-defined objects in view. I am convinced that if it became apparent that material benefit will be a natural consequence of membership no beating of the drum will be necessary for recruiting purposes, a remark which applies perhaps with especial force to Ireland. There has been an immense amount of vain talk and wrangling about the number of representatives on this board and on that, about lay and professional controllers, as though a layman and a professional man, or woman, differed in their make-up. They are exactly similar in all the essentials. Hath not a layman eyes? Hath not a layman hands, organs, dimensions, senses, affections, passions?—fed with the same food, hurt with the same weapons, subject to the same diseases, healed by the same means, warmed and cooled by the same winter and summer as a professional man is? If you prick him, will he not bleed? If you tickle him, does he not laugh? If you poison him, does he not die?* These wranglings may interest and absorb the big-wigs, but the plain work-a-day nurse is unconcerned. What she wants are a little more money, a little less labour, a little more rest, and an outlook for her time of disablement through old age or infirmity. After all, within these are comprehended the ambition of every toiler—the little heaven that does not appear to be unattainable in this life. Wherever any or all of these incidents of betterment have been realised in other spheres it has been through co-operation, will-power, organisation. In this great Empire of ours, in which hundreds of thousands of nurses are engaged, and in which there is, doubtless, scope for many thousands more, there is no reasonable limit to what may be achieved. And on this account the Imperial ideal and the educational ideal embodied in the College propaganda are *par excellence*, and should be thoroughly and energetically developed. Each of these deserves the dignity of special staffs of workers at headquarters. The College should have its branches in every country where waves the British flag, and there should be recognised media for the dissemination of cheap and useful professional literature, as well as for lectures, the holding of examinations, and the awarding of distinctions. I have especially in my mind the young Irish nurse who emigrates. What a boon to her to find on her arrival a little coterie of friends, members of the same society, engaged at the same life work. In her new home she finds at once a link with the home she has left.

Am I justified in suggesting a plan of campaign? I feel convinced that if the true note is struck it will find an echo in many unexpected quarters. Party leaders will become submerged in a movement that finds no room for cliques or cranks. It is a workers' cause, and the workers' wants are easily defined. Let us make straight for the goal.

IERNE.

* Apologies to the "Merchant of Venice."

